# Development of a LC-MS/MS Method for the Simultaneous Detection of Tricarboxylic Acid Cycle Intermediates in a Range of Biological Matrices

**DOI:** 10.1155/2017/5391832

**Published:** 2017-09-18

**Authors:** Omar Al Kadhi, Antonietta Melchini, Richard Mithen, Shikha Saha

**Affiliations:** ^1^Food and Health Programme, Quadram Institute Bioscience, Norwich NR4 7UA, UK; ^2^Department of Urology, Norfolk and Norwich University Hospitals NHS Foundation Trust, Norwich NR4 7UY, UK

## Abstract

It is now well-established that perturbations in the tricarboxylic acid (TCA) cycle play an important role in the metabolic transformation occurring in cancer including that of the prostate. A method for simultaneous qualitative and quantitative analysis of TCA cycle intermediates in body fluids, tissues, and cultured cell lines of human origin was developed using a common C18 reversed-phase column by LC-MS/MS technique. This LC-MS/MS method for profiling TCA cycle intermediates offers significant advantages including simple and fast preparation of a wide range of human biological samples. The analytical method was validated according to the guideline of the Royal Society of Chemistry Analytical Methods Committee. The limits of detection were below 60 nM for most of the TCA intermediates with the exception of lactic and fumaric acids. The calibration curves of all TCA analytes showed linearity with correlation coefficients *r*^2^ > 0.9998. Recoveries were >95% for all TCA analytes. This method was established taking into consideration problems and limitations of existing techniques. We envisage that its application to different biological matrices will facilitate deeper understanding of the metabolic changes in the TCA cycle from in vitro, ex vivo, and in vivo studies.

## 1. Introduction

**Figure 1:**
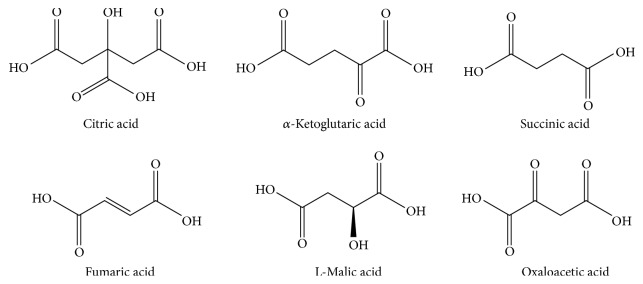
Chemical structure of TCA cycle intermediates.

The tricarboxylic acid (TCA) cycle, also known as Krebs' cycle, represents a crucial metabolic pathway in almost all living organisms [1]. It encompasses a cyclic series of reactions that oxidises citrate to generate NADH (reduced form of nicotinamide adenine dinucleotide, NAD) and a series of metabolites that can be used for amino acid and lipid synthesis, as well as the regeneration of citrate through the condensation of acetyl CoA with oxaloacetate (Figure 1) [2]. NADH feeds into oxidative phosphorylation to generate adenosine-triphosphate (ATP) [3]. In certain cell types and pathological conditions citrate is exported from mitochondria as opposed to being oxidised [4, 5]. For example, healthy prostate epithelial cells have a modified TCA cycle that favours the export of citrate out of the mitochondrion to provide an energy source for spermatozoa [6]. Likewise, many cancer cells favour the export of citrate for lipid synthesis [7]. The different metabolic fate of citrate between healthy and cancer cells has been the focus of attention of several research groups, and has been studied as possible adjunct to diagnosis and a target for cancer therapy [8–12]. Many of published reports on the analyses of intermediates of the TCA cycle, including citrate, have used enzymatic or Nuclear Magnetic Resonance (NMR) methods for their detection and quantification [5, 13–15]. Studies that used enzymatic methods often relied on spectrophotometric methods and thus provide indirect measurement of citrate and related TCA cycle intermediates [16–18]. NMR offers the advantage of requiring minimal sample preparation but lacks the sensitivity in comparison to liquid chromatography (LC) [19]. Analysis of small molecules using targeted liquid chromatography tandem mass spectrometry (LC-MS/MS) is thought to provide higher sensitivity especially from matrices where compounds or molecules are at low concentrations [20, 21]. Our aim was to develop a LC-MS/MS method that would allow the simultaneous detection of TCA cycle intermediates in different biological matrices of human origin with a relatively easy sample preparation and high sensitivity. Previous studies have described the application of liquid chromatographic methods coupled with mass spectrometry for the determination of specific intermediates of the Krebs cycle (e.g., succinic acid, citric acid) in body fluids [22, 23]. The simultaneous determination of TCA intermediates has been described in natural waters or bacterial cultures [24–26], but there are only few reports in samples of human origin including tumour specimens [27–29]. This study reports the development and validation of an extraction method from human plasma, urine, prostate tissue, and cultured cells that allows the qualitative and quantitative analysis of TCA cycle intermediates by using LC-MS/MS. It was envisaged that the simultaneous detection and quantification of these key metabolites by LC-MS/MS could be a useful research tool and may provide a more holistic picture of the changes that occur in the TCA cycle in different cancer types including prostate cancer.

## 2. Experimental

### 2.1. Chemicals and Reagents

Tricarboxylic acid standards [glutamic acid (≥99%), citric acid (≥99%), isocitric acid (≥93%), *α*-ketoglutaric acid (≥99%), malic acid (≥99%), oxaloacetic acid (≥97%), succinic acid (≥99%), and fumaric acid (≥99%)] were purchased from Sigma® (Sigma-Aldrich Company Ltd., Dorset, UK). Lactic acid (≥85%) was also obtained from Sigma (Sigma-Aldrich Company Ltd., Dorset, UK). Deuterated D4-citric acid (2,2,4,4-D4, 98%) was purchased from Cambridge Isotope Laboratories, Inc. (CK Isotopes Ltd., Leicestershire, UK). Synthetic urine control sample and fatty acid-free human serum albumin (HSA) were purchased from Sigma (Sigma-Aldrich Company Ltd., Dorset, UK). Trichloroacetic acid and perchloric acid were purchased from Sigma (Sigma-Aldrich Company Ltd., Dorset, UK). Water was obtained from a Milli-Q® Integral Water Purification System (Millipore Limited, Hertfordshire, UK).

### 2.2. Human Body Fluids

For purposes of method development and with local ethical approval, urine (*n* = 5) and plasma (*n* = 10) samples were obtained from healthy subjects. Plasma samples (10 *μ*l) were mixed with 5% trichloroacetic acid (80 *μ*l) and 0.1 mg/ml internal standard (deuterated D4-citric acid, 10 *μ*l). The mixture was vortexed for 30 seconds and kept on ice for 5 minutes (vortex twice/2 minutes). Samples were centrifuged at 17,000 ×g for 10 minutes (4°C) and supernatants were transferred to HPLC vials for analysis by LC-MS/MS. After filtration by using a minisart Sterile-ED 0.20 *μ*m filter, urine samples (10 *μ*l) were added to 0.2% formic acid (80 *μ*l) and 0.1 mg/ml internal standard (deuterated D4-citric acid, 10 *μ*l). The mixture was vortexed for 30 seconds and kept on ice for 5 minutes (vortex twice/2 minutes). Samples were then centrifuged at 13,000 ×g for 5 minutes (4°C). Supernatants were transferred to HPLC vials and analysed by the LC-MS/MS on the same day.

### 2.3. Human Prostate Tissue

Frozen samples of histologically proven benign (*n* = 5) and cancer (*n* = 5) prostate tissue were obtained from the Norwich Biorepository (Norfolk and Norwich University Hospital Human Tissue Bank). Benign tissue samples were obtained from patients undergoing radical prostatectomy for organ confined prostate cancer. The protocol was approved by the Faculty of Medicine and Health Science Research Ethics Committee in January 2013 (Reference: 2012/2013-37). Tissue samples were kept at −80°C until pulverized with liquid nitrogen using a tissue grinder (BioPulverizer, Stratech Scientific Limited, Newmarket, UK). 500 *μ*l of 3 M perchloric acid was added to each 20 mg of tissue powder and homogenised using an automated tissue homogeniser (IKA Ultra-Turrax T8 Disperser, Fisher Scientific Ltd, Loughborough, UK) for 1 minute on medium speed. The homogenate was left on ice for 10 minutes and then centrifuged at 12,000 ×g for 10 minutes at 2–4°C. Supernatants were transferred to HPLC vials and analysed by the LC-MS/MS on the same day.

### 2.4. Human Cell Lines

Human cancerous prostate adenocarcinoma (PC3) cells were purchased from European Collection of Authenticated Cell Cultures (ECACC) (number 90112714). Cells were routinely cultured as monolayers in Ham's F-12 medium supplemented with 10% foetal bovine serum (FBS) in a humidified atmosphere containing 5% CO_2_ at 37°C. The culture medium was changed every two days. Cells were grown to 80% confluency on 10 cm dishes before performing the extraction of the TCA cycle intermediates. Briefly, cells were washed twice with 10 ml 0.9% sodium chloride (Sigma-Aldrich Company Ltd., Dorset, UK) and were harvested using 0.025% (w/v) trypsin (Gibco®, Fisher Scientific Ltd, Loughborough, UK) in a phosphate-buffered saline solution (D-PBS w/o Calcium Magnesium) (Fisher Scientific Ltd, Loughborough, UK). After cell count with a haemocytometer, cell suspensions were centrifuged at 1,200 ×g for 10 minutes at room temperature. After discarding the supernatant, cells were gently suspended by adding 10 ml of 0.9% sodium chloride before centrifugation at 1,200 ×g for 10 minutes. The supernatant was then removed and 0.5 ml of perchloric acid (0.3 mM) was added. The mixture was kept on ice for 10 minutes followed by further centrifugation at higher speed of 12,000 ×g for 10 minutes. Supernatant was then transferred to HPLC vials and kept at −20°C until required for analysis. LC-MS/MS analysis was carried out within 24–72 hours from sample collection. All reagents were kept on ice (below 6°C) at all times. The concentration of all TCA analytes was normalised per 10^6^ cells. TCA cycle intermediates were also extracted in culture media. Briefly, the culture medium was collected before harvesting PC3 cells with trypsin and 10% of perchloric acid (3 mM) was added. Samples were kept on ice for 10 minutes followed by further centrifugation (12,000 ×g for 10 minutes) as described for cell pellets.

### 2.5. LC-MS/MS Analysis

TCA standards were reconstituted in mobile phase (0.2% formic acid) to prepare 1 mg/ml stock solutions. All stock solutions were prepared daily just before running samples in the LC-MS/MS system. A standard curve of each analyte (glutamic acid, citric acid, isocitric acid, *α*-ketoglutaric acid, malic acid, oxaloacetic acid, succinic acid, fumaric acid, and lactic acid) was produced from stock solutions (1 mg/ml) in the relevant matrix (synthetic urine, 5% fatty acid-free HSA, cell culture medium acidified with 0.2% formic acid). 100 *μ*l of each standard was mixed in a total volume of 1 ml to obtain a mixed standard solution (100 *μ*g/ml each standard). A five-point standard curve was produced with a 10-fold serial dilution from the highest concentration to 0. 10 *μ*l of deuterated D4-citric acid was added to all samples as an internal standard (final concentration of 10 *μ*g/ml) to allow quantification based on the ratio of the internal standard to each intermediate peak. Agilent 1200 series LC 6490 Triple Quad LC-MS mass spectrometer was used (Agilent Technologies, CA, US). Phenomenex® Luna C-18, Kinetex PFP, Kinetex XB-C18, and Kinetex-C18 columns were used to achieve an optimal separation of all TCA intermediates and to obtain good peak shapes. The flow rate was 0.4 ml/minute with a mobile phase of 0.2% formic acid. Electrospray ionisation (ESI) was used in the positive mode (ESI^+^) for glutamic acid and in the negative mode (ESI^−^) for the other TCA cycle intermediates. 2 *μ*l was used for the injection volume and the autosampler was maintained at 4°C. The Agilent 6490 Triple Quad LC-MS/MS system comprised a degasser, binary pump, cooled autosampler, column oven, and 6490 mass spectrometer. The gas temperature was 200°C and flow was 16 l/minute; sheath gas temperature was 300°C with flow of 11 l/minute and nebuliser pressure of 50 psi. Capillary voltage was 3500°C and 3000°C for positive and negative polarity, respectively. The LC eluent flow was sprayed into the mass spectrometer interface without splitting. TCA cycle intermediates were monitored by tandem MS using multiple reaction monitoring (MRM) mode. Identification was achieved based on retention time and product ions.  Table 1 summarises the monitored ions and the optimised MS operating parameters of the analytes.

### 2.6. Analytical Validation

#### 2.6.1. Linearity

Calibration curves were obtained by using authentic standards (glutamic acid, citric acid, isocitric acid, malic acid, succinic acid, fumaric acid, and lactic acid) spiked in a matrix containing deuterated D4-citric acid as internal standard. Ratio of analyte and internal standard peak area was plotted against the corresponding concentration to obtain the calibration curve.

#### 2.6.2. Sensitivity

LOD and LOQ were calculated by injection of diluted solutions of TCA intermediates in each matrix. LOD was estimated as the concentration of TCA intermediates that generated a peak with an area at least 3 times higher than the baseline noise. LOQ was calculated at a signal to-noise ratio 10 times higher than the baseline noise of these compounds.

#### 2.6.3. Precision and Accuracy

To assess within run precision, plasma samples (*n* = 10) were analysed and the precision of retention time and concentration was assessed. The bioanalytical precision and accuracy of this method were calculated by analysing the same samples on 5 days for interday precision.

#### 2.6.4. Carry-Over Effect

Agilent 1200 series high performance autosampler with an injection program was used to minimise carry-over effects. Carry-over was assessed by injecting acidified water after an injection of the highest concentration of the TCA standards.

#### 2.6.5. Extraction Recovery and Matrix Effect

The matrix effect was assessed by using the postextraction spike method as indicated by RSC guideline for LC-MS measurements [30]. The same concentration of TCA intermediates was spiked in two matrices: (1) 0.2% formic acid in water to assess the LC-MS/MS method and (2) acidified synthetic urine (urine analysis) or 5% fatty acid-free HAS (plasma analysis) to assess the matrix effect with deuterated D4-citric acid as internal standard.

### 2.7. Data Analysis

Data files were explored and analysed using MassHunter Quantitative B.06 Workstation software (Agilent Technologies, CA, US). The peak area of each analyte was determined, and the concentration of the analyte was calculated using the peak area ratio (peak area of analyte/peak area of the internal standard).

## 3. Results

### 3.1. Optimisation of Mass Spectroscopy Conditions

The automated Agilent MassHunter Optimiser software was used to obtain precursor and products ions in each analyte. The collision energy was used from 0 to 80 by 10 CE step increment in negative polarity mode for all analytes except for glutamic acid in positive polarity. The fragmentor value was constant, 380 V. Decarboxylation and/or water elimination are the most intense fragmentation patterns for most of the organic acids [25]. The precursor and product ions produced by the Agilent 6490 mass spectrometer were comparable with these patterns. However, in the current study we observed selective fragments which can be useful if further identification is needed. For example, both citric and isocitric acids show a main product ion with* m/z* 111 corresponding to [M-H-CO_2_-2 H_2_O]^−^; however, isocitric acid also gives rise to a relatively stable product ion with* m*/*z* 155 corresponding to the neutral loss of two water molecules. The fragment* m/z* 155 produced by isocitric acid is hardly seen in the fragmentation pattern of citric acid. The same fragment 155 from isocitric acid was also observed by Bylund and colleagues [24]. The fragmentations results are summarised in Table 1.

### 3.2. Optimisation of LC Parameters

Waters Acquity UPLC HSS C18 and BEH Amide columns were used to achieve an optimal separation of all TCA products by using different mobile phases at different pH values. Most of the TCA cycle intermediates have shown tailing peaks. We found that the use of a Kinetex-C18 1.7 *μ*m (100 × 2.1 mm) column and guard column from Phenomenex allows good separation and peak shapes using isocratic mobile phase 0.2% formic acid in water.

### 3.3. Method Validation

The method was validated against published acceptance criteria for linearity, accuracy, precision, recovery, and sample stability [30]. We performed method validation procedures in plasma samples by choosing a matrix that would minimise the interference of endogenous compounds [31]. Due to the high levels of TCA analytes, mainly citric acid, endogenously present in human plasma, 5% fatty acid-free HAS was used for matrix match calibration with the addition of deuterated D4-citric acid as internal standard. Validation was not performed for *α*-ketoglutaric and oxaloacetic acids due to their instability.

#### 3.3.1. Linearity and Sensitivity

Calibration curves were linear over a wide range of concentrations, and the least- squares regression calibration curve was *r*^2^ = 0.999 (Table 2). LOD and LOQ values are also shown in Table 2.

#### 3.3.2. Precision and Accuracy

Intraday precision was evaluated by replicate (*n* = 10) analysis of a single human plasma sample. The precision was calculated from the relative standard deviation. The CV (%) was less than 10% for all analytes. Interday precision was evaluated by analysing the same sample by the same extraction and LC-MS/MS methods (*n* = 5 days). The CV (%) was <15% for most of the analytes except for succinic acid (18.9%). The higher CV (%) value for succinic acid can be explained by low plasma concentration of this TCA intermediate. Precision and accuracy data are presented in Table 2.

#### 3.3.3. Carry-Over Effect

One of the most commonly encountered problems in the quantification of metabolites in biological samples by LC-MS/MS technique is carry-over of tested compounds. Several parts of an injection system can contribute to carry-over effect such as needle outside, needle inside, needle seat, sample loop, seat capillary, and injection valve. In our study, we used Agilent 1200 series high performance autosampler with an injection program to minimise carry-over effects. Response for all TCA analytes was approximately zero. Only glutamic acid showed carry-over effect (<1%).

#### 3.3.4. Extraction Recovery and Matrix Effect

The recovery of the proposed method was assessed by spiking known amounts of TCA intermediates in two matrices. The matrix effect/ion suppression of all TCA intermediates was <5%, and recoveries were >95%.

#### 3.3.5. Stability

Stock solutions of TCA standards were freshly prepared before each LC-MS/MS run. However, analysis was repeated with stock solutions kept at −20°C for 7 days, and no changes were observed. This is in accordance with a study carried out by Keevil and colleagues reporting that citric acid was stable at −20°C for 30 days [23]. The stability of extracted samples was assessed following 90 injections of the same sample at 4°C. The CV (%) value was <10% for all the biological matrices tested in this study.

### 3.4. Analysis of TCA Cycle Intermediates in Human Plasma Samples

**Figure 2:**
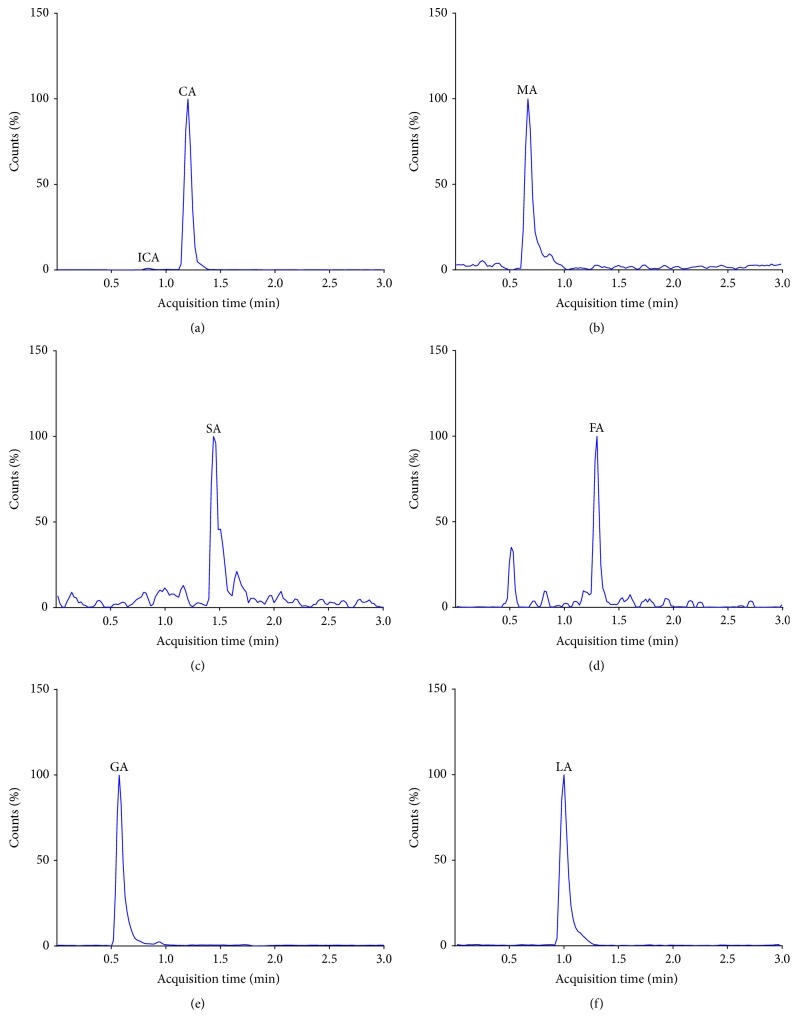
LC-MS/MS chromatograms of TCA cycle intermediates in human plasma. (a) Citric (CA) and isocitric (ICA) acids; (b) malic acid (MA); (c) succinic acid (SA); (d) fumaric acid (FA); (e) glutamic acid (GA); (f) lactic acid (LA).

Fatty acid-free HSA was used for matrix match calibration with the addition of deuterated D4-citric acid standard as internal standard to enable the simultaneous identification and quantification of TCA cycle intermediates in human plasma (*n* = 10). The calibration curve of each compound was linear, and the correlation coefficient ranged from 0.9996 to 1.0 in the range of concentrations listed in Table 2.  Figure 2 shows the detection of TCA analytes in human plasma. The calibration curve of citric acid was linear in the range of concentrations of 0–520 *μ*M. The average concentrations obtained from plasma samples (143 ± 39.1 nM) were comparable to known reference ranges of human circulating citrate (100–300 nM) [32, 33]. Previous studies confirmed that citrate with an average concentration of 135 *μ*M represents the most abundant intermediate of the TCA cycle in human blood [34].

### 3.5. Application of This Method to Other Biological Matrices of Human Origin

#### 3.5.1. Analysis of TCA Cycle Intermediates in Human Urine Samples

**Figure 3:**
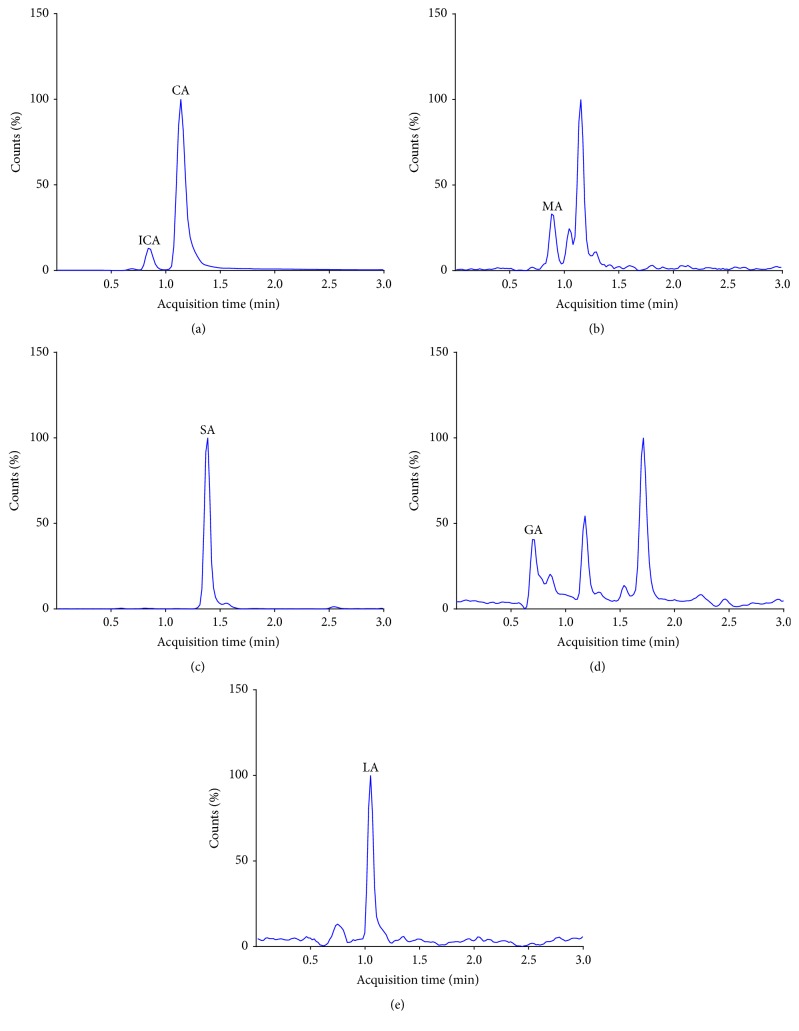
LC-MS/MS chromatograms of TCA cycle intermediates in human urine. (a) Citric (CA) and isocitric (ICA) acids; (b) malic acid (MA); (c) succinic acid (SA); (d) glutamic acid (GA); (e) lactic acid (LA).

A synthetic urine sample was used for matrix match calibration with the addition of deuterated D4-citric acid internal standard to enable quantification. Following 10-fold dilution with 0.2% formic acid, TCA cycle intermediates were successfully detected in human urine samples (*n* = 5). The calibration curve of each compound was linear, and the correlation coefficient ranged from 0.9998 to 0.9999 in the linear range of concentrations of 0–520 *μ*M. An example of detected TCA analytes in human urine is shown in Figure 3. The average level of citric acid in urine was 369.3 ± 37.1 *μ*g/ml. Previous reports have indicated that urinary citrate excretion ranged between 350 and 1,200 mg/day in healthy subjects [35, 36]. Data obtained were comparable to the reported range of urinary citrate excretion in humans taking into account the fact that the normal range for 24-hour urine volume is 800 to 2,000 ml per day.

#### 3.5.2. Analysis of TCA Cycle Intermediates in Human Prostate Tissue

**Figure 4:**
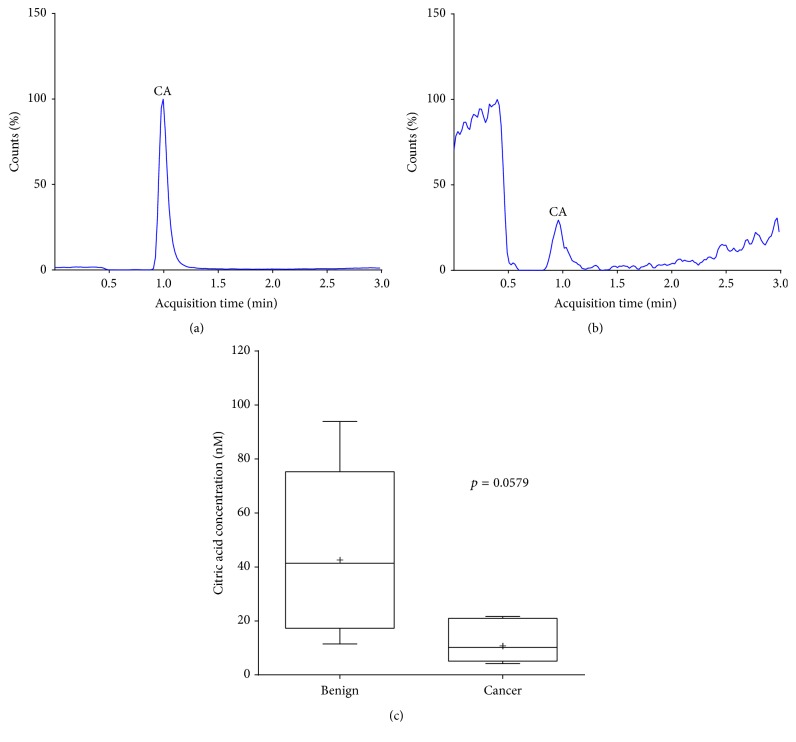
Identification and quantification of citric acid (CA) in human prostate tissue. (a) LC-MS/MS chromatogram of citric acid (CA) in benign tissue. (b) LC-MS/MS chromatogram of citric acid (CA) in cancer tissue. (c) Average concentrations (nM) of citric acid in benign and cancer tissue. Frozen tissue samples were ground to powder before subsequent extraction. Citric acid (nM) was quantified from benign (*n* = 5) and cancer (*n* = 5) prostate tissue samples using LC-MS/MS. The results are corrected for tissue weight. Mean values are shown as “+.” Statistical analysis was performed with Student's *t*-test (*p* = 0.0579).

Histologically proven cancerous and benign prostate tissue samples were obtained from the Norwich Biorepository (NNUH) in accordance with local regulatory approvals. Tissue originated from either radical prostatectomy/cystoprostatectomy specimens or transurethral resection of the prostate (TURP). Citrate was successfully detected and quantified by LC-MS/MS in both benign (*n* = 5) and cancer (*n* = 5) tissue (Figure 4). The concentration of citrate found in this study was comparable with that of published data [12].

#### 3.5.3. Analysis of TCA Cycle Intermediates in Human Cultured Cell Lines

**Figure 5:**
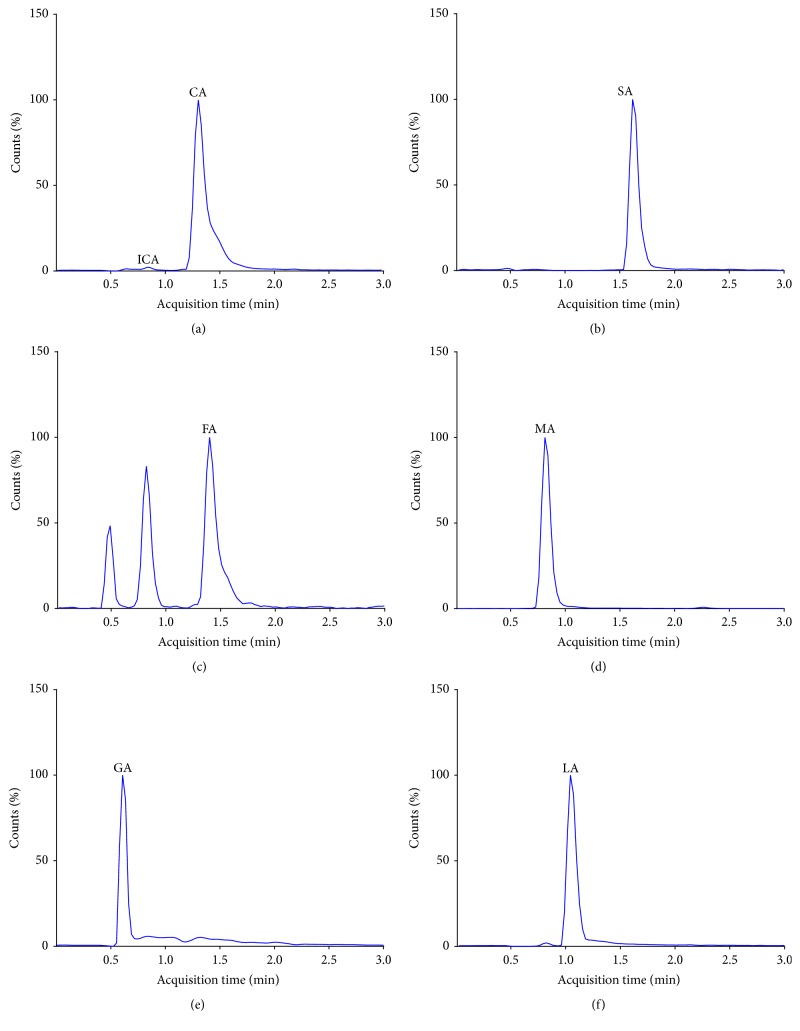
LC-MS/MS chromatograms of TCA cycle intermediates extracted from human cultured PC3 cells. (a) Citric (CA) and isocitric (ICA) acids; (b) succinic acid (SA); (c) fumaric acid (FA); (d) malic acid (MA); (e) glutamic acid (GA); (f) lactic acid (LA).

For the purpose of method development PC3, malignant androgen-independent prostate cells were cultured as previously described. Most of the TCA cycle intermediates were successfully detected and quantified using standard solutions. The calibration curve of each compound was linear and the correlation coefficient ranged from 0.96 to 1.0 in the same linear range of concentrations used for human plasma. Some of the TCA intermediates were more pH sensitive and very unstable such as oxaloacetic and *α*- ketoglutaric acids; both of these organic acids were difficult to detect and quantify. LC- MS/MS chromatograms for TCA intermediates detected from PC3 cell lysates are presented in Figure 5. Furthermore, TCA intermediates including citric, isocitric, malic, and succinic acids were successfully detected in culture media of PC3 cells (data not shown).

## 4. Discussion

The metabolite profiling of tissue and body fluids has become increasingly important to study fundamental aspects of human health and disease, as well as understanding the influence of different factors, such as drugs, diet, and lifestyle changes [37]. The use of LC-MS/MS methods for the quantification of metabolites from biological matrices enables accurate detection of target compounds in relatively low concentrations, reducing interference [20, 38]. When examining the TCA cycle intermediates, LC-MS/MS is arguably superior to enzymatic assays that invariably rely on fluorescence techniques to measure concentrations of substrates indirectly and often only allow measurement of one compound at a time [39]. The LC-MS/MS method described in this study allows simultaneous measurement of the majority of TCA cycle intermediates with relatively easy sample preparation. In addition, it allows measurement of lactic and glutamic acids which provide key information regarding the processes of glycolysis and glutaminolysis, both of which influence the concentration of metabolites that are used as substrates in the TCA cycle reactions. In a previous report describing LC-MS/MS quantification of TCA cycle intermediates from soil samples, Bylund and colleagues outlined the retention times and ion mass/charge ratio (*m/z*) for each of these intermediates [24]. Authors used ion exclusion divinylbenzene column with API3000 mass spectroscopy and the run time of this method was 30 minutes. We achieved a good separation by using a Kinetex C18 reversed-phase column with Agilent 6490 mass spectroscopy in a more time effective manner (run time = 3 minutes). Thus, the proposed method is very fast without compromising the performance of chromatographic separation. The fragmentation patterns were similar to the previous study carried out by Bylund et al. [24]. These fragmentations patterns were used to optimise the method for a wide range of biological matrices (human plasma, urine, prostate tissue, and cell lines). The extraction technique and mobile phase were modified to successfully measure more TCA intermediates in a single run. Our method has significantly improved detection and run time resulting in high sensitivity and relatively short run times. In addition, the two isomers citric and isocitric acids were successfully separated. The achieved separation is very important for quantification purposes, because both acids have the same transitions. We also observed another fragmentation pattern by analysing malic acid, which gave a main product ion 115 and 71. However, the same transition 115/71 was also produced by fumaric acid. Therefore, selective retention times are crucial for the analysis of these two TCA intermediates which were successfully separated by applying our method. However, there are some limitations in measuring *α*-ketoglutaric and oxaloacetic acids which were difficult to detect, in particular, in cultured cells. This is likely due to their instability and pH sensitivity, which has been previously reported [25, 40]. However, *α*-ketoglutaratic acid was successfully detected in some plasma samples, but it was not quantified due to the instability of the synthetic standard. Further work will be required to develop a method based on carbon-labelled standards for detecting *α*-ketoglutaric and oxaloacetic acids as already described by Rabinowitz in* Escherichia coli *and primary human fibroblast cultures [41].

To summarise, we report a LC-MS/MS method by which it is possible to successfully detect TCA cycle intermediates from a variety of biological sources. Preliminary data confirmed that this method could be also applied for the measurement of TCA intermediates in other human prostate cell lines including human normal prostatic epithelial cells (data not shown). No matrix effect was observed in different matrices due to the sample dilution and low injection volume. Measurement of citric acid, in particular, is shown to be reproducible and comparable to the ranges already reported in published literature [12]. In the last few decades, citrate has been the most studied of these intermediates in relation to prostate metabolism and has often been used as a surrogate for the function of the TCA cycle [34]. It has also been reported that citrate plasma levels are the highest among TCA cycle intermediates in humans [34], and for this reason citrate metabolism is often studied to better understand the role of the TCA cycle in the development of human metabolic diseases [33, 42]. The application of this LC-MS/MS method, which has been successfully developed for analysing human samples of different sources in a fast and reliable way, could represent a valuable analytical tool for future research.

## 5. Conclusions

Measuring TCA cycle intermediates using LC-MS/MS is feasible and allows simultaneous detection and accurate quantification of multiple compounds in different biological matrices. In this paper, we report a fully validated method which offers several advantages in particular in terms of easy sample preparation without derivatization steps, high sensitivity, and short run times. The potential application of this method for analysing the TCA cycle in a wide range of biological matrices could be important in in vitro, ex vivo, and in vivo studies.

## Figures and Tables

**Table 1:** LC-MS/MS parameters of each analyte.

Analyte	Retention time (min)	Precursor ion (*m/z*)	Product ion (*m/z*)	Collision energy	Cell accelerator voltage	Polarity
D4-citric acid	1.13	195	114	12	4	Negative
D4-citric acid	1.13	195	177	10	4	Negative
Isocitric acid	0.85	191	155	10	5	Negative
Citric acid	1.13	191	111	10	5	Negative
Citric acid	1.13	191	87	18	5	Negative
Glutamic acid	0.6	148	130	8	4	Positive
Glutamic acid	0.6	148	84	12	4	Positive
Malic acid	0.7	133	115	10	5	Negative
Malic acid	0.7	133	71	14	5	Negative
Succinic acid	1.6	117	99	10	5	Negative
Succinic acid	1.6	117	73	10	5	Negative
Fumaric acid	1.3	115	71	10	5	Negative
Fumaric acid	1.3	115	41	18	5	Negative
Lactic acid	1.05	89	43	10	4	Negative
*α*-Ketoglutaric acid	1.03	145	101	10	5	Negative
*α*-Ketoglutaric acid	1.03	145	57	10	5	Negative
Oxaloacetic acid	0.58	131	87	10	5	Negative
Oxaloacetic acid	0.58	131	41	54	5	Negative

**Table 2:** Validation data for each analyte in human plasma.

Analyte	Linearity range (*µ*M)	*R* ^2^	Precision intraday (%) (*n* = 10)	Precision interday (%) (*n* = 5)	LOD^*∗*^	LOQ^*∗*^
Citric acid	0–520	0.9998	4.9	6.3	0.06	0.18
Isocitric acid	0–520	0.9997	4.6	10.5	0.06	0.18
Malic acid	0–372	0.9999	8.9	13.5	0.06	0.18
Lactic acid	0–1100	0.9987	4.44	9.9	1	3
Succinic acid	0–840	0.9998	8.1	18.9	0.06	0.18
Fumaric acid	0–430	0.9998	8.8	12.2	1	3
Glutamic acid	0–340	0.9998	7.4	12.6	0.05	0.15

^*∗*^Values in *µ*M for 1 : 10 sample dilution.
